# GPR37 and GPR37L1 differently interact with dopamine 2 receptors in live cells

**DOI:** 10.1016/j.neuropharm.2018.11.009

**Published:** 2019-07-01

**Authors:** E. Hertz, L. Terenius, V. Vukojević, P. Svenningsson

**Affiliations:** Department of Clinical Neuroscience, Karolinska Institute, Stockholm, Sweden

**Keywords:** GPR37, GPR37L1, Dopamine 2 receptor, Pramipexole, 4-PBA fluorescence cross-correlation spectroscopy

## Abstract

Receptor-receptor interactions are essential to fine tune receptor responses and new techniques enable closer characterization of the interactions between involved proteins directly in the plasma membrane. Fluorescence cross-correlation spectroscopy (FCCS), which analyses concurrent movement of bound molecules with single-molecule detection limit, was here used to, in live N2a cells, study interactions between the Parkinson's disease (PD) associated orphan receptor GPR37, its homologue GPR37L1, and the two splice variants of the dopamine 2 receptor (D2R). An interaction between GPR37 and both splice forms of D2R was detected. 4-phenylbutyrate (4-PBA), a neuroprotective chemical chaperone known to increase GPR37 expression at the cell surface, increased the fraction of interacting molecules. The interaction was also increased by pramipexole, a D2R agonist commonly used in the treatment of PD, indicating a possible clinically relevance. Cross-correlation, indicating interaction between GPR37L1 and the short isoform of D2R, was also detected. However, this interaction was not changed with 4-PBA or pramipexole treatment. Overall, these data provide further evidence that heteromeric GPR37-D2R exist and can be pharmacologically modulated, which is relevant for the treatment of PD.

This article is part of the Special Issue entitled ‘Receptor heteromers and their allosteric receptor-receptor interactions’.

## Highlights

• GPCR interaction is studied with fluorescence cross-correlation spectroscopy.
• Interaction between GPR37 and both isoforms of D2R is detected in live cells.
• GPR37's homologue GPR37L1 is detected to interact with D2RS in live cells.
• GPR37-D2R interaction is increased by D2-like agonist and 4-PBA treatment.

## 1 Introduction

G Protein-Coupled Receptors (GPCRs) are the most common drug targets (Hauser et al., 2018) and orphan GPCRs are considered an unexploited source for future therapies (Smith, 2015). G Protein-Coupled Receptor 37 (GPR37) and G Protein-Coupled Receptor 37 like 1 (GPR37L1) are two orphan receptors which have been proposed as druggable and both are almost exclusively expressed in the central nervous system (Smith, 2015). These two receptors share more than 60% amino acid similarity, particularly in the transmembrane domains (Smith, 2015). Although the role of GPR37L1 in health and disease is sparse, mice lacking GPR37L1 showed a precocious cerebellar development (Marazziti et al., 2013) and increased seizure susceptibility (Giddens et al., 2017). Several studies have reported that GPR37 is implicated in the pathogenesis of Parkinson's disease (PD) (Kitao et al., 2007; Murakami et al., 2004; Wang et al., 2008), the second most common neurodegenerative disorder that is characterized by dopaminergic cell death. Misfolding of GPR37 causes endoplasmic reticulum stress leading to cell death (Imai et al., 2001; Kitao et al., 2007). However, if GPR37 is correctly folded and trafficked to the plasma membrane, it can exert neuroprotective actions (Lundius et al., 2013). Previous co-immunoprecipitation experiment showed that GPR37 interacts with dopamine 2 receptor (D2R), and thereby becomes enriched at the cell surface (Dunham et al., 2009). D2R is widely expressed in the brain and is a key therapeutic target in routine PD treatment. However, at present, it is not known whether GPR37 interacts with the different D2R isoforms. D2R has two splice forms, long (D2RL) and short (D2RS). D2RL is mainly expressed on the postsynaptic membrane and D2RS functions as an autoreceptor on presynaptic nerve terminals (Usiello et al., 2000).

The dynamic local interactions between receptors within their plasma membrane microdomain are now known to be essential in determining the final intracellular communication in response to pharmacological treatments (Briddon and Hill, 2007). In addition, GPCR heterodimerization is both time and space dependent (Borroto-Escuela et al., 2017; Briddon et al., 2018). For these reasons, receptor-receptor interactions are preferably studied in live cells, where the membrane structure is preserved. Furthermore, novel spectroscopy methods allow both spatial and temporal resolution of GPCR interaction in live cells (Briddon et al., 2018). One such technology is fluorescence cross correlation spectroscopy (FCCS), which detects concurrent molecular movement that occurs only when the examined molecules are bound to one another.

To, in live cells, further investigate GPR37 interactions with both D2R isoforms, we used FCCS. We also studied the interactions between GPR37L1 and both D2R isoforms. In order to evaluate whether interactions between GPR37 or GPR37L1 and D2R isoforms are altered when receptor surface densities are increased, the cells were treated with 4-phenylbutyrate (4-PBA), a neuroprotective chemical chaperone which traffics GPR37 to the cell surface (Kubota et al., 2006). Furthermore, we examined the effects of pramipexole, a D2R-like agonist which is commonly used in the clinics for the treatment of PD, on the hetero-receptor interactions.

## 2 Materials and methods

### 2.1 Molecular cloning

Human GPR37 and GPR37L1 (Origene #RG208054 and #RG208132 respectively) were amplified using PCR with Phusion polymerase (ThermoFisher, Waltham, MA, USA). The primers used are available upon request. Products were subcloned into a p-EGFP-N1-vector for expression as fusions to the N-terminus of the enhanced Green Fluorescent protein (eGFP) (gift from Prof. Xu) using EcoRI and XhoI (New England Biolabs, Ipswich, MA, USA) and ligated with T4 ligase according to manufacturer's instructions. The plasmids were transformed by heat shock into TOPO10 E. Coli (ThermoFisher, Waltham, MA, USA). Full receptor sequence and correct reading frame were confirmed with Sanger sequencing. D2RS-Td-tomato and D2RL-Td-tomato were generated similarly and confirmed with Sanger sequencing.

### 2.2 Cell culture

Neuro 2a (N2a) cell line from mouse, obtained from ATCC (CCL-131™ Mus musculus brain neuroblastoma), were grown in DMEM with high glucose, 10% FBS, 1% penicillin-streptomycin, 2 mM L-Glutamine and 1 × Non-Essential Amino Acids (NEAA). N2a cells stably expressing GPR37-eGFP were generated by transfecting the plasmids into wild type N2a cells (WT-N2a) using Lipofectamine 2000. The cells were transfected in OPTI-MEM, and the cell culture medium was replaced 4 h after transfection. 24 h after transfection selection with 500 μg/ml geneticin was initiated. After plasmid-free cells have died, single clones were selected and thereafter, grown in media with 100 μg/ml geneticin. Despite several attempts conducted in parallel with the generation of stably transformed GPR37-eGFP expressing N2a cells (GPR37-eGFP-N2a), no stable N2a cell line expressing GPR37L1-eGFP could be established.

For transient expression of GPR37L1-eGFP and/or D2R isoform tagged with Td-Tomato, WT-N2a and GPR37-eGFP- N2a cells were seeded in 8-well chambers with cover slide bottom (Nunc Lab-Tek II #155409) at a density of 1.5 × 10^4^ cells per well. The following day the cells were transfected with 250 ng of GPR37L1 and/or 350 ng of specified D2R isoform DNA containing plasmids using 0,7 μl Lipofectamine 2000 per well. The cells were incubated for 4–6 h and the cell culture medium was changed to phenol red-free DMEM with additives as described above. In untreated cells, FCCS measurements were initiated 24 h after transfection. Before experimental start, the cell culture medium was changed to serum free DMEM Flourobrite to minimize background fluorescence. Pramipexole dihydrochloride (Sigma Aldrich, MO, USA), freshly dissolved in PBS, or PBS alone, were added to assigned wells. FCCS measurements started directly after pramipexole addition. To enhance GPR37 trafficking to the plasma membrane, 4-phenylbutyrate (4-PBA, Sigma Aldrich, MO, USA) was added to the cell culture medium at a total concentration of 1 mM and the cells were grown for 48 h. Pramipexole was thereafter added as described above. If not stated otherwise all cell culture products mentioned were from ThermoFisher, Waltham, MA, USA.

### 2.3 FCCS measurements

**Fig. 1:**
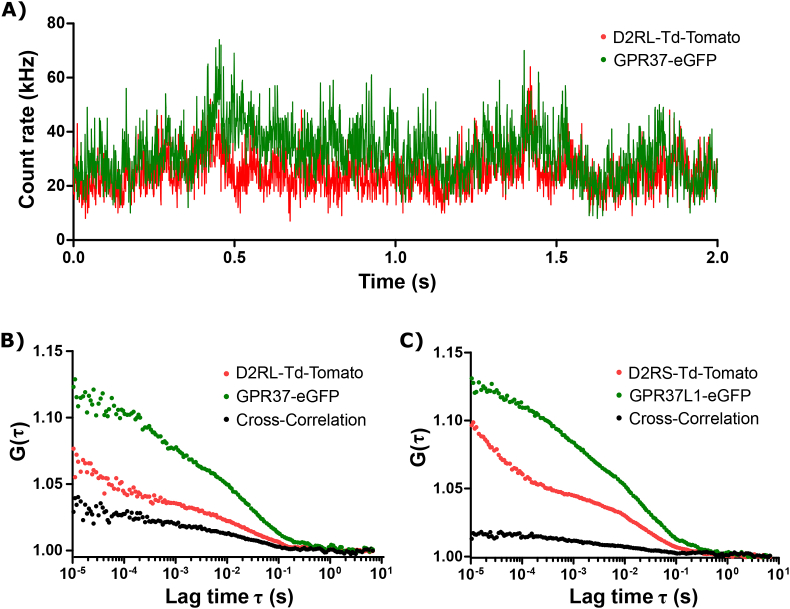
FCCS measurements. A) Fluorescent fluctuations detected over time in two separate channels showing correlating peaks. B) Auto- (green and red) and cross-correlation (black) curves showing the lateral dynamics and interactions between GPR37-eGFP (green) and the long isoform D2RL-Td-Tomato (red). C) Auto- (green and red) and cross-correlation (black) curves showing the lateral dynamics and interactions between GPR37L1-eGFP (green) and D2RS-Td-Tomato (red).

Fluorescence cross-correlation spectroscopy (FCCS) analyses fluctuations in fluorescence intensity, that originate through random motion of spectrally distinct fluorescent molecules, to quantitatively characterize their concentration, diffusion and interactions between them (for in-depth theoretical background, please see references (Bacia et al., 2006; Weidemann and Schwille, 2013)). In particular, simultaneous occurrence of fluctuation peaks from two spectrally distinct fluorophores are analyzed by temporal cross-correlation analysis to identify co-diffusion, indicating interaction between the examined molecules (Fig. 1A). The amplitude of the cross correlation curve is directly proportional to the degree of binding (Fig. 1B and C) (Bacia et al., 2012).

FCCS measurements were performed using an upright LSM510 ConfoCor3 instrument (Carl Zeiss, Jena, Germany) with a water immersion 40× objective, NA 1.2 equipped with two silicone avalanche photodiodes (SPCM AQR-1x, PerkinElmer LifeSciences) to enable single molecule detection. Excitation of eGFP was exerted with a 488 nm line of an Ar/ArKr laser (1% transmission, 50% of full operation power) while Td-Tomato was excited with a 543 nm He/Ne laser (7% transmission). Pinhole size in front of the detector was 70 μm. Emitted light was separated from incident light by a main dichroic beam splitter HFT 488/543 and spectrally split with a secondary dichroic beam splitter, NFT 545. In front of the detectors, bandpass filters, BP 505-530 and LP 580 respectively, further narrowed the detected spectrum. FCCS measurements were performed at the apical plasma membrane above the virtually transparent cell nucleus, identified by a vertical fluorescence intensity scan. Fluorescence intensity fluctuations were recorded in a series of 10 consecutive 20-s-long measurements. Occasionally recorded deviating traces (due to immediate bleaching, bright vesicles or lost signal) were omitted and the average curve comprising the remaining measurements was analyzed.

All measurements were performed at controlled temperature (37 C°) in humid air containing 5% CO_2_ using a heated microscope stage (Heat Insert P, PeCon GMBH) and an atmosphere controlling unit (CTI-Controller 3700 digital, PeCon GMBH).

### 2.4 Temporal auto- and cross-correlation analysis

The autocorrelation function G(τ) is defined as:

(1) $$G\left(\tau \right)=1+\frac{\langle \delta I\left(t\right)\times \delta I\left(t+\tau \right)\rangle }{\langle I\left(t\right)\rangle^{2}},$$

where τ is the lag time, the chevron brackets denote time average 〈I(t)〉, δI(t) is the intensity difference from the average intensity 〈I(t)〉 at a time point t and δI(t+τ) is the intensity difference at t+τ. G(τ) plotted as a function of τ yields the experimentally derived autocorrelation curve (Fig. 1B and C).

The cross-correlation function G_CC_(τ) is defined as:

(2) $$G_{CC}\left(\tau \right)=1+\frac{\langle {\text{δI}}_{green}\left(\text{t}\right)\times {\text{δI}}_{red}\left(\text{t}+\text{τ}\right)\rangle }{\langle {\text{I}}_{green}\left(\text{t}\right)\rangle \times \langle {\text{I}}_{red}\left(\text{t}\right)\rangle }$$

Auto- and cross-correlation analysis were performed using the dedicated ConfoCor3 running software.

The relative cross-correlation amplitude (RCCA), which is proportional to the number of dually labeled receptor-receptor complexes, was calculated as the amplitude of the cross-correlation curve, normalized to the amplitude of the autocorrelation curve recorded for the green channel, (Bacia et al., 2012). To avoid single time point noise, the RCCA was determined as an average over 20 consecutive points, beginning at τ = 1.05 × 10^−5^. Each RCCA data point represents a single-cell measurement. Since RCCA measures the proportion of red molecules (i.e. D2R) bound to green molecules (i.e. GPR37), the RCCA will appear to be low if the green molecules are out-numbered in the plasma membrane. Therefore, to directly compare the extent of receptor-receptor binding between different cells, the ratio between the number of red- and green-tagged receptor molecules was similar in all cells analyzed. To minimize crosstalk between the channels, the number of eGFP-tagged receptors was always lower than the number of Td-Tomato-tagged receptors. Rogacki et al. has reported RCCA detected in the cytoplasm for eGFP linked to Td-Tomato, i.e. 100% binding as positive control, to 0.75 ± 0.04 and eGFP separate from td-Tomato, i.e. 0% binding as negative control, to 0.10 ± 0.08 (Rogacki et al., 2018).

### 2.5 Confocal laser scanning microscopy (CLSM) imaging

The multitrack acquisition mode was used for CLSM imaging. In the imaging mode, the main dichroic beam splitter, HFT 488/543/633, the secondary dichroic beam splitter NFT 545, and the bandpass filters BP505-530 and LP 580 were used. Scanning speed was 25,6 μs/pixel in a 1024×1024 pixel frame. The images were acquired using avalanche photodiodes without averaging. Image post processing was not performed. Only contrast and brightness, applied to the entire picture, were changed.

### 2.6 Statistics

Data are presented as mean ± SD. Unpaired *t*-test was used to compare groups. Samples were analyzed for outliers using Grubbs test, however, exclusion/inclusion of outlier data did not change the main findings regarding significant differences. Data was evaluated for normal distribution and equal variances between the groups. The statistical analysis was performed using GraphPad Prism 5.04. Effects were considered significant when p < 0.05.

## 3 Results

### 3.1 GPR37 interacts with the long (D2RL) and the short (D2RS) isoforms of the D2R

**Fig. 2:**
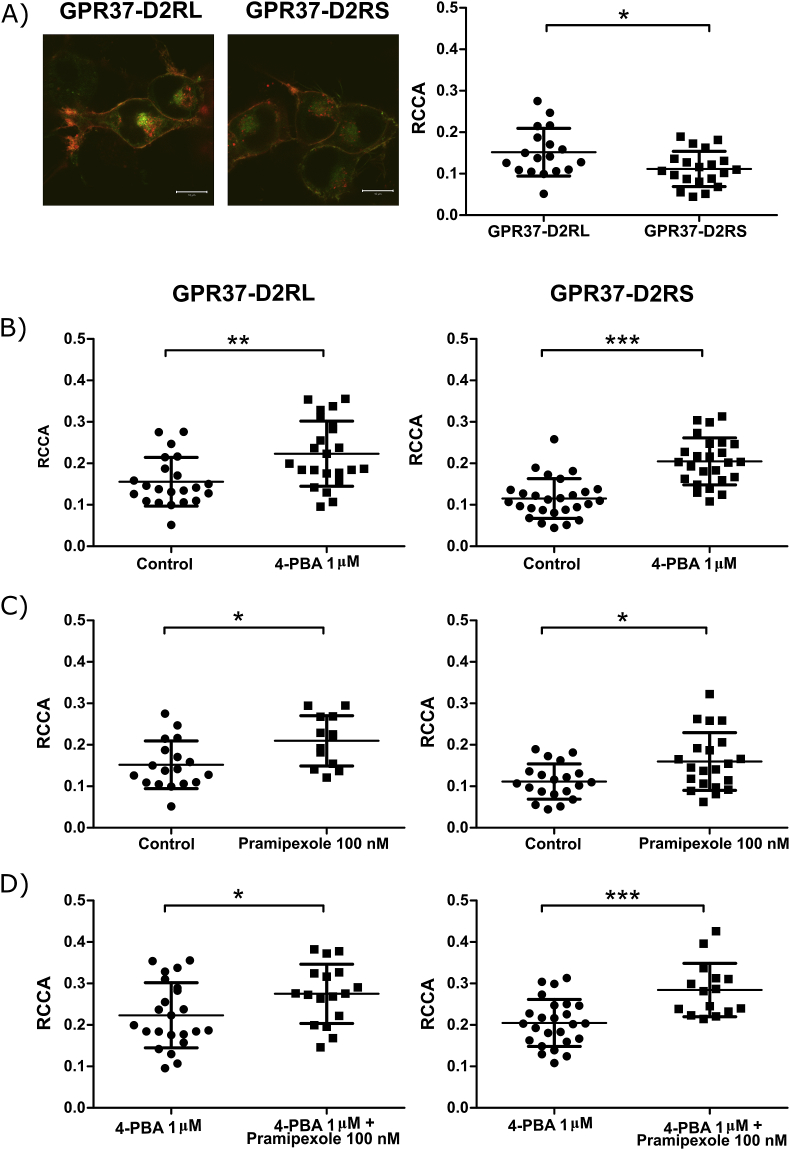
Quantification of GPR37-eGFP interactions with the long (D2RL-Td-Tomato) or the short (D2RS-Td-Tomato) isoform of the D2R by RCCA analysis. A) CLSM image of N2a cells expressing GPR37-eGFP (green) and D2RL-Td-Tomato (red) *(left panel*) and GPR37-eGFP (green) and D2RS-Td-Tomato (red) (*middle panel),* scale bar 10 μM. RCCA comparison between D2RL-Td-Tomato and D2RS-Td-Tomato in complex with GPR37-eGFP (*right panel*). B) RCCA reflecting GPR37-D2RL interactions (*left panel*) and GPR37-D2RS (*right panel*) after 48 h of treatment with 1 μM 4-PBA. C) RCCA reflecting GPR37-D2RL interactions (*left panel*) and GPR37-D2RS (*right panel*) in cells 24 h after transfection, immediately after treatment with 100 nM pramipexole. D) RCCA reflecting GPR37-D2RL interactions (*left panel*) and GPR37-D2RS (*right panel*) first treated with 1 μM 4-PBA for 48 h and then measured immediately after addition of 100 nM pramipexole. The RCCA values are means ± SD from 14 to 25 cells. *p < 0,05, **p < 0,01, ***p < 0,001 by two tailed *t*-test.

Time series were initially performed with GPR37 and both isoforms of D2R. Representative auto- and cross-correlation curves showing interaction between GPR37-eGFP and D2RL-Td-Tomato is seen in Fig. 1B. In untreated cells, the RCCA is low, indicating that only a small fraction of interacting molecules are present or the signal is due to detection of bleed through. D2RL-transfected cells, however, showed a somewhat higher proportion of interacting molecules compared to D2RS-transfected cells which under these conditions display RCCA in the same range as the negative control reported by Rogacki (Fig. 2A). It has been reported that D2R, although unspecified whether long or short isoform, facilitates GPR37 trafficking to the plasma membrane (Dunham et al., 2009). We hypothesized that the low interaction frequencies in GPR37-D2RL and GPR37-D2RS cells may be due to the low level of plasma membrane localization of GPR37 in heterologous systems (Dunham et al., 2009). Therefore, we treated the cells with 1 μM 4-PBA, a chemical chaperone known to facilitate GPR37 trafficking to the plasma membrane (Mimori et al., 2012). The average RCCA increased in both the D2RL and D2RS transfected group (Fig. 2B).

### 3.2 GPR37-D2R interaction is stimulated by dopamine D2-like agonist treatment

The effect of a dopamine D2-like agonist on the GPR37-D2R interaction was investigated. Pramipexole, a D2/D3 agonist, was chosen as it is currently used for the treatment of PD in the clinics. Directly after the addition of 100 nM pramipexole, RCCA increased in vehicle treated cells (Fig. 2C). The same effect of pramipexole was seen in 4-PBA treated cells (Fig. 2D).

To reduce the risk of bleed through, sequential illumination was performed in a subset of the cells where only one laser was used at one time. Even though sequential illumination reduced the quality of the data, a cross-correlation between GPR37 and D2RL was still detected. Furthermore, it was shown that pramipexole treatment increased the cross-correlation between GPR37 and D2R. Autocorrelation amplitudes of two channels, td-Tomato and eGFP, were not changed (data not shown).

### 3.3 GPR37L1 interacts with D2RS isoform

**Fig. 3:**
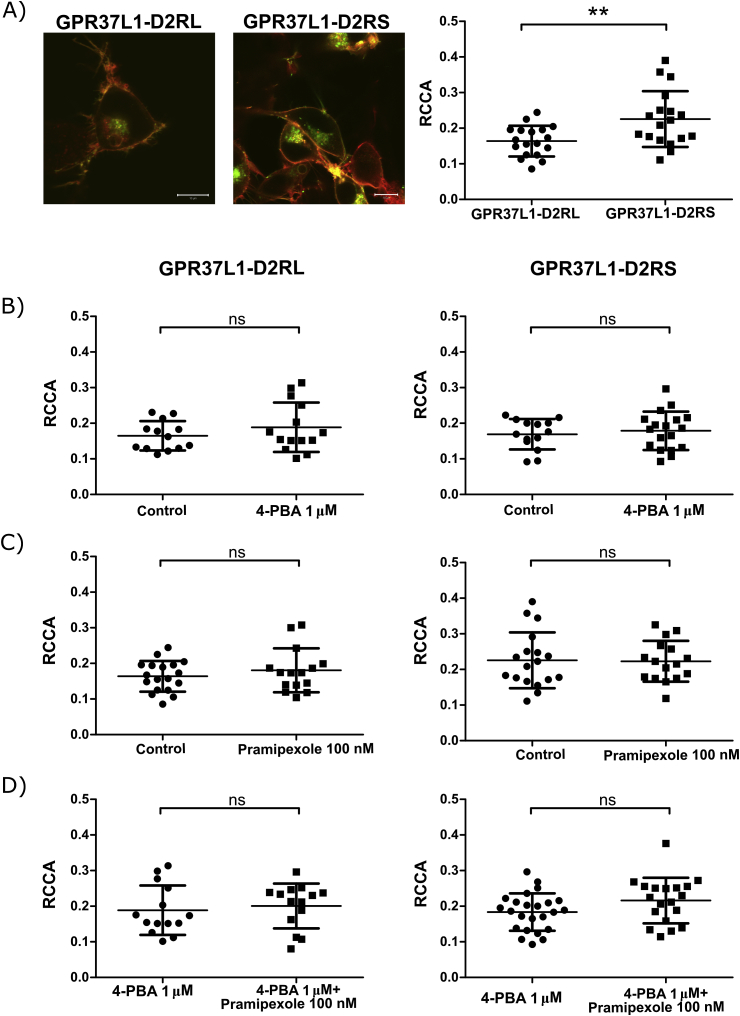
Quantification of GPR37L1-eGFP interactions with the long (D2RL-Td-Tomato) or the short (D2RS-Td-Tomato) isoform of the D2R by RCCA analysis. A) *Left panel*: CLSM image of N2a cells expressing GPR37L1-eGFP (green) and D2RL-Td-Tomato (red) (*left panel)* and GPR37L1-eGFP (green) and D2RS-Td-Tomato (red) (*middle panel),* scale bar 10 μM. RCCA comparison between D2RL-Td-Tomato and D2RS-Td-Tomato in complex with GPR37L1-eGFP (*right panel)*. B) RCCA reflecting GPR37L1-D2RL interactions (*left panel*) and GPR37L1-D2RS (*right panel*) after 48 h of treatment with 1 μM 4-PBA. C) RCCA reflecting GPR37L1-D2RL interactions (*left panel*) and GPR37L1-D2RS (*right panel*) in cells 24 h after transfection, immediately after treatment with 100 nM pramipexole. D) RCCA reflecting GPR37L1-D2RL interactions (*left panel*) and GPR37L1-D2RS (*right panel*) first treated with 1 μM 4-PBA for 48 h and then measured immediately after addition of 100 nM pramipexole. The RCCA values are means ± SD from 13 to 24 cells. *p < 0,05, **p < 0,01, ***p < 0,001 by two tailed *t*-test.

WT-N2a cells transiently transfected to express both GPR37L1-eGFP and either D2RL or D2RS, showed, in agreement with a previous study (Dunham et al., 2009), a high level of GPR37L1 at the cell surface (Fig. 3A). GPR37L1 and D2RS showed cross-correlation, indicating simultaneous movement through the plasma membrane (Fig. 1C). In contrast to GPR37-D2R interaction studies, RCCA was higher in D2RS than in D2RL with GPR37L1 (Fig. 3A). The treatment of 4-PBA and/or pramipexole did not change the fraction of interacting GPR37L1-D2R molecules (Fig. 3B–D).

## 4 Discussion

In the present study, the interactions between GPR37, or its close homologue, GPR37L1, and D2Rs were examined using FCCS in live cells. GPR37 interacts more strongly with the long isoform of D2Rs, while GPR37L1 has a preference for the shorter D2R isoform. In addition, the clinically used D2R-like agonist pramipexole specifically increased the fraction of interacting GPR37-D2R molecules, regardless of isoform type. This effect was, however, not seen with GPR37L1, indicating a specific modulatory role of pramipexole on GPR37-D2R interactions.

The formation of heterodimers affects several properties of a GPCR, such as correct trafficking and fine tuning of signaling (Milligan, 2007). Co-expression of GPR37 with D2R has previously been shown to enhance GPR37 trafficking to the plasma membrane (Dunham et al., 2009). Importantly, GPR37 has been suggested to have dual roles in PD pathogenesis depending on the cellular localization (Lundius et al., 2013), raising the importance of receptor trafficking. The results from the present study support the existence of GPR37-D2R heterodimers in live cells. In the previous studies, it was not indicated which D2R splice form was used for their experiments. Here, we showed a higher level of GPR37-D2RL than GPR37-D2RS in naïve N2a cells. One possible explanation for this phenomenon may be that postsynaptic D2RL isoform traffics GPR37 to the membrane at a higher extent under baseline conditions.

Due to low expression of GPR37 at the cell surface in cell lines, several methods or conditions have been developed to increase trafficking to the plasma membrane including differentiation (Lundius et al., 2014), protein-protein interaction (Dunham et al., 2009) and use of chaperones such as 4-PBA (Kubota et al., 2006). GPR37-eGFP-cells treated with 4-PBA for 48 h increased the RCCA, possibly due to the translocation of GPR37 to the plasma membrane which enables interaction. 4-PBA seems to shift GPR37 from intracellular ER accumulation to a functional membrane expression, here shown by responding to dopamine agonist treatment as expected. Translocation of aggregating proteins is a desired feature in the treatment of neurodegenerative disorders as several are linked to improper protein aggregation.

Several lines of evidence suggest that GPR37 modulates dopamine signaling, both in PD and in other dopamine-linked disorders, such as bipolar disorder and major depressive disorder (Tomita et al., 2013). Treatment with dopamine D2R-like agonists is a clinical routine in PD management, although the spectrum of therapeutic and adverse effects is not yet fully understood. In the current study, the treatment with one of the most widely prescribed dopamine agonists, pramipexole, increased the interaction between GPR37 and D2R. In a previous study, it was shown that the co-expression of GPR37 and D2R increased D2R ligand binding affinity. However, it was uncertain whether the D2R agonist regulated the interaction between GPR37 and D2R or not (Dunham et al., 2009). The concentration of pramipexole, 100 nM corresponding to 28.4 ng/ml, can be physiologically relevant. In serum from PD patients treated with pramipexole, the concentration is 3–5 ng/ml at steady state (Eisenreich et al., 2010). However, in a study of with a high dose of pramipexole in Alzheimer's disease the mean concentration in the cerebral spinal fluid was 498 ng/ml (Bennett et al., 2016). Further studies, particularly in intact brain slices at the striatal level, are needed to elucidate the functional impact of the GPR37-D2R interaction, determine the lowest effective dose of pramipexole and clarify the effect of agonist induced interaction. Interestingly, several reports demonstrated a neuroprotective effect of pramipexole *in vitro* and *in vivo* models of PD (Kim et al., 2015; Li et al., 2010; Motyl et al., 2018). Moreover, Gu and colleagues specifically showed that the neuroprotective effect of pramipexole was not dependent on dopamine receptors (Gu et al., 2004). Further studies are required to determine whether the observed neuroprotective effect of pramipexole could be due to increased interaction of GPR37 with D2R. From a clinical perspective, the pramipexole induced D2R-mediated G*i*-signaling in the indirect striatopallidal pathway could be augmented by the GPR37/D2R interaction as GPR37 is also reported to be G*i* coupled (Meyer et al., 2013; Zheng et al., 2018). Such an increased inhibition of the striatopallidal neurons might improve movements in patients with PD.

This is the first time that an interaction between GPR37L1 and D2R has been demonstrated. For D2RL, the RCCA is low and, in contrast to the GPR37-D2R interaction, there was no effect of 4-PBA or pramipexole treatment to induce increased RCCA. The average RCCA level is higher than the negative control but this level might vary due to different experimental set ups. However, since the bleed through is expected to be unchanged between different D2R isoforms, which were measured with the same experimental set up, the higher RCCA detected for GPR37-D2RS should be due to simultaneous movements.

Both GPR37 and GPR37L1 generate a RCCA, indicating a co-diffusion with D2R. However, GPR37 and GPR37L1 respond differently to 4-PBA and/or pramipexole treatments. The largest differences between GPR37 and GPR37L1 lie in the extracellular N-terminus and in the intracellular C-terminus tail, the two likely targets for the drug treatments. In the current study, only small differences in RCCA were detected between the pre- and postsynaptic splice variants of D2R and in this system no differences in treatment responses is detected between the two isoforms. The transmembrane domains and the intracellular loops are the major fractions involved in the dimerization of GPCRs (Milligan, 2007). D2RL and D2RS are generated by alternative splicing, in which there is only a 28 amino acid difference between the two types in the large third intracellular loop. The transmembrane sequences of GPR37 and GPR37L1 are highly preserved. Hence, the small differences might be due to highly similar transmembrane domains between GPR37 and GPR37L1, which is responsible for the interaction. Furthermore, *in vivo*, heterodimerization is determined by cellular expression of the receptor isoform in addition to their possibility to interact.

FCCS is increasingly being used to study molecular interactions as this approach enables one to study interactions in live cells with both spatial and temporal resolution. Recently, dimerization by agonist treatment was evaluated by FCCS (Petersen et al., 2017). However, as for all methods, there are limitations. One of the major limitations of FCCS is the bleed through between channels. It gives rise to a false cross-correlation, and it is difficult to exclude its occurrence. However, by comparing RCCA, the differences detected are corrected for eGFP intensity differences. To further examine and limit the bleed through in naïve cells, sequential illumination was used. Nevertheless, a cross-correlation was still detected suggesting that the cross-correlation detected is not due to cross-talk. Another possibility for misinterpretation of RCCA changes after treatment is that the drug treatment itself change the pH and subsequently the fluorescent properties of eGFP or Td-Tomato. However, since both 4-PBA and pramipexole treatment results in changes in GPR37, but not in GPR37L1, expressing cells, the effects seen are not due to changes in fluorescent properties. Lastly, as FCCS detects simultaneous movement, not direct interaction, meaning that functional complexes which diffuse together can result in cross-correlation even if the proteins are not directly binding to each other. Dunham et al. showed direct interaction between GPR37 and D2R by co-immunoprecipitation in transient transfected HEK-293 cells (Dunham et al., 2009). In addition, in a yeast two hybrid screen, Sokolina et al. detect an interaction between D2RL and GPR37 (Sokolina et al., 2017). It is also noteworthy, that both GPR37 (Morato et al., 2017; Sokolina et al., 2017) and D2R (Fernandez-Duenas et al., 2015) interacts with adenosine A_2A_ receptor, and it has been proposed that a functional GPR37/D2R/A_2A_R-complex is able to fine-tune the effects of pharmacological treatment (Morato et al., 2017; Sokolina et al., 2017). Although the current study demonstrates a functional interaction between GPR37 and D2R, as FCCS detects only the simultaneous movement of molecules, it is difficult to confirm, nor exclude, the existence of oligo-trimer GPR37/D2R/A_2A_R complexes.

## 5 Conclusion

Increasing evidence emphasize protein-protein interactions as central for receptor function in health and disease. Using FCCS we can, with single molecule sensitivity, detect and quantitatively analyze, the interaction between GPR37 and both isoforms of D2R directly in the plasma membrane. The fraction of interacting molecules is increased by the neuroprotective chaperone 4-PBA, which ensures GPR37 localization in the plasma membrane. Furthermore, the interaction is increased by treatment with the D2R-like agonist pramipexole, suggesting a previously unknown effect of treatment with this dopamine agonist and highlighting the clinical relevance of the interaction in Parkinsonism. In addition, we report an interaction between GPR37L1 and D2RS which is not affected of either treatment. These data broaden the molecular evidence that dynamic GPR37-D2R complexes affects dopamine modulation and reinforce the importance of future studies in intact neuronal circuitries.

## Declaration of interest

None.
